# Vistas in Signaling Pathways Implicated in HSV-1 Reactivation

**DOI:** 10.3390/ijms252212472

**Published:** 2024-11-20

**Authors:** Kostas A. Papavassiliou, Amalia A. Sofianidi, Fotios G. Spiliopoulos, Vassiliki A. Gogou, Athanasios G. Papavassiliou

**Affiliations:** 1 First University Department of Respiratory Medicine, Sotiria Chest Hospital, Medical School, National and Kapodistrian University of Athens, 11527 Athens, Greece; konpapav@med.uoa.gr (K.A.P.); gogvanessa@gmail.com (V.A.G.); 2 Department of Biological Chemistry, Medical School, National and Kapodistrian University of Athens, 11527 Athens, Greece; amsof.00@gmail.com (A.A.S.); spiliopoulosfotis99@gmail.com (F.G.S.)

## 1. Introduction

Ancient Greek physicians, including Hippocrates, documented skin conditions resembling herpes as early as 500 before common era (BCE), but it was not until the 1920s that Lowenstein successfully isolated the herpes virus from human lesions, significantly advancing our understanding of the infection [1]. Herpes simplex virus (HSV) types 1 and 2 are DNA viruses that lead to lifelong infections, with oral HSV-1 being among the most widespread viral infections globally [2]. The World Health Organization (WHO) estimates that approximately 3.8 billion people under the age of 50, or 64.2% of the global population, are infected with HSV-1, impacting their oral, nasal, or ocular cavities [3].

After the initial infection, HSV-1 enters a lifelong dormant state in sensory and other types of neurons. During HSV-1 latency, the viral genome remains silent in peripheral neurons, but it retains the ability to become reactivated and resume infection [4]. Although viral lytic gene expression is largely suppressed during latent infection, the latency-associated transcript (LAT) remains actively transcribed, poised to trigger reactivation when the appropriate stimulus occurs [5]. HSV-1 reactivation can be triggered by various factors, including physical or emotional stress, illness, fever, UV light exposure, or hormonal changes [6]. These triggers compromise the immune system and cause recurrent outbreaks, which can impose significant physical and psychological burdens.

**Figure 1:**
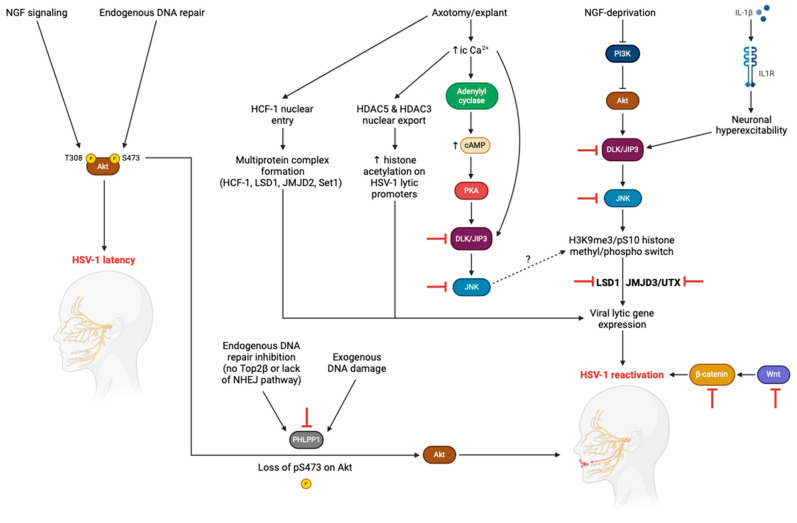
The current scenery of signaling pathways that contribute to HSV-1 reactivation. NGF deprivation potentiates the neuronal JNK stress pathway, where DLK and JIP3 stimulate JNK, leading to the phosphorylation of histone 3 at serine residue 10 that induces lytic gene expression during phase I of HSV-1 reactivation. During phase II, histone demethylases (LSD1 and JMJD3/UTX) remove repressive chromatin marks from HSV-1 lytic promoters. Neuronal hyperexcitability is a DLK-mediated trigger of HSV-1 reactivation that can be induced by upstream IL-1β release. Axotomy/explant causes a rise in intracellular calcium levels that potentiates adenylyl cyclase and DLK/JIP3, resulting in JNK activation and perhaps the same histone methyl/phospho switch that triggers viral gene expression associated with HSV-1 reactivation under NGF deprivation. Increased intracellular calcium augments histone acetylation on HSV-1 lytic promoters due to the nuclear export of histone deacetylases (HDAC5 and HDAC3). Additionally, following axotomy/explant, the transcriptional cofactor HCF-1 translocates to the nucleus and binds to immediate early viral gene promoters recruiting histone demethylases (LSD1 and JMJD2) and methyltransferases (Set1) to upregulate lytic gene expression. While physiological NGF signaling and endogenous DNA repair mechanisms maintain HSV-1 latency via Akt activation (phosphorylation), endogenous DNA repair inhibition and accumulation of exogenous DNA damage allow the Akt Ser473-specific phosphatase PHLPP1 to dephosphorylate and deactivate Akt, evoking the reactivation of the HSV-1 genome. Wnt/β-catenin signaling promotes HSV-1 reactivation probably via a β-catenin-dependent transcriptional reprogramming event that orchestrates lytic gene expression and virus shedding. Red inhibitory arrows represent points of therapeutic targeting. Akt, protein kinase B; cAMP, cyclic adenosine monophosphate; DLK, dual leucine zipper kinase; H3K9me3, histone 3 lysine 9 trimethylation; H3pS10, phosphorylation of serine 10 of histone H3; HCF-1, host cell factor-1; HDAC5, histone deacetylase 5; HDAC3, histone deacetylase 3; IL-1β, interleukin-1 beta; IL1R, interleukin 1 receptor; ic, intracellular; JIP3, JNK-interacting scaffold protein 3; JMJD2, Jumonji C domain-containing histone lysine demethylase 2; JMJD3, Jumonji C domain-containing histone lysine demethylase 3; JNK, c-Jun N-terminal kinase; LSD1, lysine-specific histone demethylase 1; NHEJ, non-homologous end joining; NGF, nerve growth factor; PHLPP1, PH domain and leucine-rich repeat protein phosphatase 1; PI3K, phosphoinositide 3-kinase; PKA: protein kinase A; Set1, histone-lysine N-methyltransferase and H3 lysine-4 specific protein; S473, serine residue 473; Top2β, topoisomerase 2β; T308, threonine residue 308; UTX, ubiquitously transcribed tetratricopeptide repeat X chromosome. This figure was created using the tools provided by BioRender.com (accessed on 1 November 2024).

Despite over a century of scientific progress, we have yet to develop a therapeutic strategy to eliminate the virus, primarily due to our incomplete understanding of the HSV-1 life cycle, especially its elusive reactivation pathways. Herein, we provide a synoptic overview of recent advancements in the signaling pathways engaged in HSV-1 reactivation and discuss current and potential therapeutic approaches to target these pathways (Figure 1).

## 2. Recent Advances in Signaling Pathways Involved in HSV-1 Reactivation

### 2.1. Wnt/β-Catenin Pathway

The Wingless (Wnt)/β-catenin pathway plays a vital role in neuronal regeneration and survival [7]. Building on this knowledge, it was demonstrated that the Wnt/β-catenin signaling pathway exhibits varying expression levels in the trigeminal ganglia during the HSV-1 latency-reactivation cycle [8]. Specifically, the investigators found a higher number of neurons expressing β-catenin in the trigeminal ganglia of mice latently infected with the wild-type HSV-1 strain compared to those infected with a LAT-null mutant virus, which lost its ability to maintain dormancy [8]. This finding suggests that β-catenin is a key factor in regulating viral latency, potentially influencing the virus’s ability to remain dormant inside the host. Nevertheless, treating latently infected neurons with iCRT14, a β-catenin inhibitor, or KYA1797K, a Wnt antagonist, led to reduced virus shedding during reactivation induced by explant, indicating that β-catenin may also be partly responsible for orchestrating transcriptional reprogramming that favors viral lytic gene expression and the release of virus progeny. This potential dual role of β-catenin in HSV-1 latency and reactivation appears to depend on cell type and context, and further studies are necessary to uncover its underpinnings.

### 2.2. JNK Cell Stress Pathway

Previous studies reported that HSV-1 latency represents a phase during which viral lytic gene expression is downregulated because the associated promoters are silenced within inactive genomic regions (heterochromatin), preventing their exposure to transcription factor and RNA polymerase binding. Thus, for HSV-1 to reactivate, its lytic promoters must be accessible in the absence of viral proteins. Cliffe et al. were the first to demonstrate how HSV-1 exploits chromatin by modulating neuronal signaling pathways to trigger reactivation. Mechanistically, a neuronal stress pathway elicited by nerve growth factor (NGF) deprivation activates the dual leucine kinase (DLK) and c-Jun N-terminal kinase (JNK)-interacting scaffold protein (JIP3), which induces the activation of JNK. JNK enters the nucleus and attaches to HSV-1 lytic promoters, and instead of recruiting histone demethylases to remove repressive methylation marks, such as histone 3 lysine 9 trimethylation (H3K9me3) and H3K27me3, it phosphorylates serine 10 of histone H3 (H3pS10), which neutralizes the adjacent repressive heterochromatin marks. This process is termed methyl/phospho switch and upregulates viral lytic gene expression during phase I (i.e., the initial wave of gene expression) of reactivation [9]. Complementing these findings, a recent study revealed that the transcription factor c-Jun, the main downstream effector of the neuronal JNK stress pathway, promotes the progression to full HSV-1 reactivation and is not implicated in the initiation of phase I lytic gene expression mediated by JNK [10]. Furthermore, during the initial infection of neurons by HSV-1, c-Jun establishes a reactivation-competent form that responds to specific stress cues. This suggests that, following initial HSV-1 infection, c-Jun signaling leaves a molecular imprint which reshapes the epigenetic landscape of both viral and host genomes. Another trigger that depends on the neuronal JNK stress pathway to evoke HSV-1 reactivation is neuronal hyperexcitability. Stress, UV exposure, or fever trigger the release of interleukin-1β, which increases neuronal excitation and potentiates JNK stress signaling that could induce HSV-1 reactivation [11].

HSV-1 is also reactivated after mechanical injury to human neurons, and researchers in the field of HSV biology have developed experimental models of HSV reactivation in response to axotomy/explant. Axotomy stimulates a rise in calcium within the neurons, which activates both adenylyl cyclase and DLK, leading to the upregulation of JNK. It remains to be revealed whether JNK signals downstream using the same mechanism to bring about reactivation as in the context of neuronal stress affecting viral chromatin. High levels of intracellular calcium also ignite a widespread increase in the acetylation of histones because of the nuclear export of histone deacetylases (HDACs) 5 and 3, explaining the upsurge of acetylated histones detected on the lytic promoters of HSV-1 after axotomy/explant. Additionally, explant-induced reactivation is augmented by the activity of the transcriptional coregulator host cell factor-1 (HCF-1), which undergoes nuclear translocation and tethers to the viral promoters of immediate early (IE) genes, including *ICP27*, *ICP4*, and *ICP0*, partly acting to upregulate early (E) genes. To initiate lytic gene expression, HCF-1 recruits the lysine-specific histone demethylase 1 (LSD1) and Jumonji C domain-containing histone lysine demethylase 2 (JMJD2) [6].

### 2.3. DDR and Akt Pathway

Persistent growth factor signaling through the Akt/mechanistic target of the rapamycin (mTOR) pathway plays an important role in achieving HSV-1 latency in neurons [12]. HSV latency is maintained by NGF signaling; however, when the NGF receptor, tropomyosin receptor kinase A (TrkA), is blocked, it inhibits the phosphoinositide 3-kinase (PI3K)/Akt/mTOR complex 1 (mTORC1) pathway, resulting in Akt dephosphorylation and triggering HSV-1 reactivation [12]. Hu et al. recently revealed that DNA damage response (DDR) signaling interacts with the Akt pathway to regulate HSV-1 latency. Their findings indicate that endogenous DNA double-strand breaks, generated by the topoisomerase 2β-DNA cleavage complex (TOP2βcc), are critical for activating Akt/mTORC1 signaling and HSV-1 latency maintenance [13]. This occurs via affecting the phosphorylation status and subcellular localization of Akt. When endogenous DNA repair mechanisms become inhibited or exogenous DNA damage accumulates, Akt activation is compromised, and HSV-1 reactivates its genome. In the case of endogenous DNA repair inhibition, Akt phosphorylation at serine 473 is not maintained, while in the setting of excessive exogenous DNA damage, Akt is dephosphorylated by the Akt Ser473-specific phosphatase PH domain and leucine-rich repeat protein phosphatase 1 (PHLPP1) and retained at nuclear DNA damage sites [13].

## 3. Targeting Signaling Pathways in HSV-1 Reactivation

Given the widespread prevalence of HSV-1 infections and the recurring episodes of viral reactivation, innovative strategies to manage this condition are urgently needed. One promising avenue lies in targeting specific signaling pathways, aiming to disrupt the mechanisms that trigger viral reactivation. By focusing on these pathways, we can develop targeted interventions that may significantly reduce the frequency of HSV-1 reactivations, ultimately improving the quality of life for those affected.

The initial molecules identified to target the Wnt/β-catenin pathway are the β-catenin inhibitor iCRT14 and the highly potent and selective Wnt antagonist KYA1797K. Harrison and Jones demonstrated that these compounds effectively hamper productive HSV-1 infection [14]. As the Wnt/β-catenin pathway bears a crucial role in HSV-1 reactivation, these compounds hold promise not only in curbing active infections but also in potentially reducing or eliminating viral reactivation episodes. KYA1797K facilitates the assembly of the β-catenin destruction complex, leading to the ubiquitination of β-catenin [8]. iCRT14 suppresses β-catenin-dependent transcription by disrupting its interaction with T-cell factor/lymphoid enhancer factor (TCF/Lef) transcription factors and hindering the binding of TCF/Lef to DNA [14]. Given the pivotal contribution of the Wnt pathway to HSV-1 reactivation and the elevated levels of β-catenin found in neurons where the virus remains dormant, these molecules are likely to effectively interrupt the reactivation process.

Cliffe et al. [9], who suggested that HSV-1 reactivation requires the upregulation of JNK signaling, employed two JNK inhibitors (SP600125 and AS601245) that completely blocked HSV-1 reactivation in various neurons triggered by PI3K inhibition or dexamethasone, as well as by axotomy/explant of trigeminal ganglia derived from latently infected mice. Since DLK and JIP3 are key components of the neuronal JNK stress pathway and their expression is almost neuron-specific, developing drugs that target these proteins may prove to be an effective therapeutic strategy to prevent HSV-1 reactivation. As JNK signaling functions in concert with chromatin remodeling complexes to induce HSV-1 reactivation during phases I and II, targeting this pathway together with histone demethylases (e.g., LSD1, JMJD2, JMJD3, ubiquitously transcribed tetratricopeptide repeat X chromosome (UTX)) may represent a promising combinatorial therapeutic approach against HSV-1 reactivation.

Another potential therapeutic target to prevent HSV-1 reactivation is PHLPP1, which dephosphorylates and deactivates Akt when the balance between DDR and NGF signaling that maintains virus latency is distorted. Blocking PHLPP1 with small-molecule inhibitors might curb HSV-1 reactivation.

**Figure 2:**
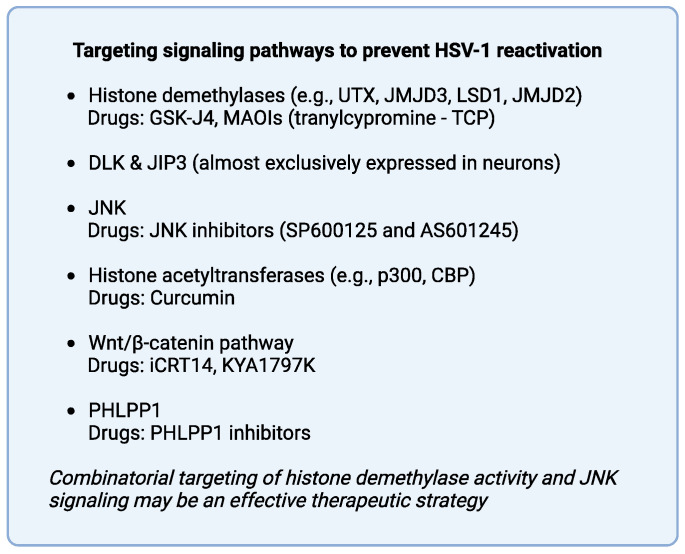
Summary of potential therapeutic targets associated with signaling pathways that promote HSV-1 reactivation. Examples of drugs that inhibit HSV-1 reactivation are included for most therapeutic targets.

All of the above therapeutic targets are host factors, and as such, there is a low probability of developing drug resistance when targeting them compared to when targeting viral proteins (Figure 1 and Figure 2). Nonetheless, considering the potential toxicity of targeting host cell functions, further research is required to unravel the precise molecular mechanisms involved and to refine targeted agents for favorable yet safe therapeutic application.

## 4. Conclusions

HSV-1 recurrence is a result of viral reactivation from a latent state within human cells prompted by various external and internal triggers. Recent studies on HSV-1 biology have considerably expanded our knowledge on the molecular control of reactivation, revealing numerous signaling pathways that promote lytic gene expression and production and shedding of progeny, including the Wnt/β-catenin pathway, the neuronal JNK stress pathway, the Akt pathway, and the epigenetic pathways exploited by HSV-1. This has provided a therapeutic opportunity allowing for the development of selective agents that target important components of signaling pathways which foster viral reactivation. Preclinical data have already identified therapeutic targets associated with HSV-1 reactivation and suggest that blocking these pathways with small-molecule inhibitors can prevent viral recurrence. Further research in this field will validate these findings and hopefully instigate their translation into clinical practice.
